# The London Panel Committee

**Published:** 1914-05-30

**Authors:** 


### The London Panel Committee.

### DOCTORS AND NURSING FOR THE INSURED.

The proceedings of the Panel Committee for London have now been opened to the professional press, and the first meeting under these conditions was held on Tuesday last. Owing to the amount of work that the Insurance Act involves, members found before them an agenda as long as that of the London Insurance Committee itself, and of a far more complicated character on account of the difficult questions that arise between Commissioners, Insurance Committee, chemists, and approved societies. Although the Committee sat for three hours, a special meeting is to be held to finish the agenda.

The Committee expressed the opinion that proprietary drugs and preparations should not be supplied at the cost of the drug fund, and that "Thermogene" should be regarded as a proprietary preparation. The most important matter before the meeting was somewhat hurriedly dealt with at its close. This was a report giving the professional view of that part of the Budget which sets aside funds to aid the establishment of laboratories and the provision of skilled nursing. The Committee discussed what assistance would be most beneficial to the practitioner and to the insured person, and decided to appoint a deputation to lay before the Chancellor and the Chairman of the Insurance Commissioners the terms upon which the services of medical referees should be available. The deputation was also to urge that chemical, pathological, and bacteriological laboratories were desirable, but that they should be administered by a central body like the London County Council, and not by the borough.

The Panel Service Sub-Committee also recommended that the following view be expressed by the deputation: "That the services of skilled nurses should be available without fee to the practitioner on the panel, for the nursing of insured persons for whom such attendance is necessary."

The Committee agreed to this, and to an amendment to add the words "and that it should be a condition attaching to the receipt of a grant that provision shall be made for increases in the salaries of nurses and for pensions on retirement."

The Sub-Committee also recommended: "That it is desirable that the present voluntary nursing agencies and societies should be aided under suitable safeguards, rather than that nurses should be appointed by the State, the local authorities, the Insurance Committees, or by approved societies."

Dr. Claude Taylor moved the following amendment: "That the conditions attaching to the receipt of a grant by a society should provide that the nurses employed shall be adequately trained, and that no religious or other test shall be required from applicants for appointment as nurses."

Dr. H. J. Cardale thought that the words in the motion, "under suitable safeguards," were sufficient without the amendment, and would prevent Biblewomen being sent to nurse, while at the same time not excluding the valuable aid received from religious nursing bodies. The amendment was lost and the recommendation agreed to.

